# Reduced bone turnover in non-obese women with polycystic ovary syndrome: a cross-sectional study on procollagen-1 N-terminal peptide and beta-C terminal telopeptide

**DOI:** 10.1590/1806-9282.20251481

**Published:** 2026-05-08

**Authors:** Mehmet Mete Kirlangic, Erdem Sahin, Mefkure Eraslan Sahin, Merve Vural Yalman, Esra Akdemir, Pınar Birol İlter, Serhan Kutuk

**Affiliations:** 1 Tuzla State Hospital, Department of Obstetrics and Gynecology – Istanbul, Turkey.; 2 Kartal Dr. Lütfi Kırdar City Hospital, Department of Obstetrics and Gynecology – Istanbul, Turkey.; 3 Kayseri City Hospital, Department of Obstetrics and Gynecology – Kayseri, Turkey.; 4 Sivas Numune Hospital, Department of Obstetrics and Gynecology – Sivas, Turkey.; 5 Kütahya City Hospital, Department of Obstetrics and Gynecology – Kütahya, Turkey.; 6 Sabuncuoğlu Şerefeddin Training and Research Hospital, Department of Obstetrics and Gynecology – Amasya, Turkey.

**Keywords:** Polycystic ovary syndrome, Bone remodeling, Vitamin D, Osteoporosis, Biomarkers, Bone resorption

## Abstract

**OBJECTIVE:**

Polycystic ovary syndrome may impair bone metabolism. The aim of this study was to assess serum type-1 collagen N-terminal procollagen and beta-C terminal telopeptide levels in non-obese women with polycystic ovary syndrome.

**METHODS:**

This cross-sectional study comprised 174 patients, 18–35 years old. Among the patients with menstrual irregularity, 82 were diagnosed with polycystic ovary syndrome according to the Rotterdam criteria and 92 were in the control group. Women with obesity (BMI>30 kg/m^2^) were excluded. Hormonal and metabolic parameters, 25-hydroxy vitamin D, procollagen-1 N-terminal peptide, and beta-C terminal telopeptide levels were measured.

**RESULTS:**

Demographics and most biochemical parameters were comparable between groups. Polycystic ovary syndrome patients had significantly lower 25-hydroxy vitamin D (80.12±68.00 vs. 117.08±73.92 ng/mL, p=0.010), procollagen-1 N-terminal peptide (597.36±501.49 vs. 863.47±509.01 ng/mL, p=0.013), and beta-C terminal telopeptide (572.88±585.94 vs. 929.91±641.38 ng/L, p=0.004) levels compared with controls.

**CONCLUSION:**

Non-obese women with polycystic ovary syndrome show significantly reduced bone formation and resorption markers, suggesting adverse effects on bone turnover. These findings suggest a low-turnover bone state in non-obese polycystic ovary syndrome women.

## INTRODUCTION

Polycystic ovary syndrome (PCOS) is the most common endocrine–metabolic disorder in reproductive age women, affecting approximately 6–15% worldwide^1^. It is characterized by hyper-androgenism, ovulatory dysfunction, and polycystic ovarian morphology. In addition to reproductive symptoms, PCOS is linked to metabolic disorders such as insulin resistance, type 2 diabetes, and cardiovascular disease^1^. Recent evidence highlights the heterogeneous nature of PCOS, with variations in menstrual patterns and hypothalamic–pituitary–ovarian axis regulation across different presentations of the syndrome^2,3^.

To standardize the assessment of bone turnover, the International Osteoporosis Foundation (IOF) and the International Federation of Clinical Chemistry and Laboratory Medicine (IFCC) recommend serum type-1 collagen N-terminal propeptide (PINP) and serum β-C-terminal telopeptide of type I collagen (βCTX) as reference biochemical markers of bone formation and resorption, respectively^4^.

Notably, 25-hydroxyvitamin D (25OH-D) is a key pro-hormone regulating calcium metabolism and influencing bone density; however, its direct association with PINP and βCTX remains uncertain, with evidence varying between observational studies and randomized trials^5^. In contrast, the anabolic effects of estrogens and androgens on bone metabolism are well-established^5^.

In PCOS, alterations in bone turnover and bone mineral density (BMD) have been reported, likely due to hormonal changes, but study results are conflicting. Recent evidence suggests that chronic low-grade inflammation and oxidative stress—both common features in PCOS—may negatively influence bone turnover by altering osteoblast and osteoclast activity. While Berberoglu et al.^6^ found no difference in bone markers and BMD between PCOS and controls, Yüksel et al.^7^ reported lower BMD in women with PCOS. More recent data suggest that BMD in PCOS varies according to body mass index (BMI). A 2024 meta-analysis demonstrated significantly lower spinal and femoral BMD in women with PCOS and BMI<27 kg/m^2^, whereas obese PCOS patients had preserved or higher BMD compared with BMI-matched controls^8^. Furthermore, recent cross-sectional findings indicate that bone turnover marker levels also differ according to BMI status in PCOS^9^.

Given these inconsistencies, the aim of the present study was to evaluate serum P1NP and βCTX levels in non-obese women with PCOS.

## METHODS

This cross-sectional study was conducted at Tuzla State Hospital, Istanbul, Turkey, according to the Declaration of Helsinki with approval from the local ethics committee (09.2021.873). All patients included in the study submitted a signed consent form.

### Study population

In this cross-sectional study, 174 consecutive patients were divided into groups as follows: 82 diagnosed with PCOS according to the Rotterdam criteria and 92 with a regular menstrual cycle (at 27–35 days for at least the last 6 months), 18–35 years old, a BMI<30 kg/m^2^ (BMI was calculated as weight ratio and height squared), and a normal examination and medical history as the control group. In the presence of obesity (BMI >30 kg/m^2^), patients were excluded because obesity affects bone health. PCOS was diagnosed when two of the following criteria were met: (1) clinical or biochemical hyperandrogenism, (2) chronic ovulatory dysfunction, or (3) polycystic ovarian morphology^8^. The exclusion criteria were as follows: (1) pregnancy, (2) BMI>30 kg/m^2^, (3) non-classical congenital adrenal hyperplasia, (4) Cushing’s disease, (5) hyperprolactinemia, (6) known thyroid disease, or (7) systemic disease. Each patient’s age, ethnicity, nulliparity, smoking habits, height, and weight were recorded at baseline. Congenital adrenal hyperplasia and Cushing’s syndrome were excluded based on patient history, clinical findings, and review of electronic hospital records. No specific hormonal stimulation or suppression tests were performed, and cases with any clinical suspicion were not included in the study.

### Sampling and data collection

After the participants fasted for 10–12 h, blood serum samples were drawn from the antecubital region during the follicular phase on the third day of the menstrual cycle. Follicle-stimulating hormone (FSH), luteinizing hormone (LH), estradiol (E2), thyroid-stimulating hormone (TSH), prolactin (PRL), total testosterone, dehydroepiandrosterone sulfate (DHEA-S), hemoglobin A1c (HbA1c), fasting blood glucose, and insulin levels were measured in the biochemistry clinic of Tuzla State Hospital. Insulin resistance (IR) was calculated using HOMA-IR and the following formula: fasting glucose (mg/dL) * fasting insulin (mIU/L)/405. An extra 4 mL of venous blood was obtained to analyze the bone turnover markers P1NP, βCTX, and 25OH-D_3_, and serum was placed in separator biochemistry tubes and centrifuged at 1,000 rpm for 10 min at 4°C. The supernatant from the samples was transferred into clean 1.5-mL Eppendorf tubes and stored at -80°C until tested with enzyme-linked immunosorbent assay (ELISA). P1NP, βCTX, and 25OH-D_3_ levels in the serum were measured using ELISA Kits (BT-LAB E3601Hu, BT-LAB E3602Hu, and BT-LAB E1543Hu). The 25OH-D_3_ assay (BT-LAB E1543Hu) was calibrated using a six-point standard curve (1–120 ng/mL), which demonstrated excellent analytical performance (quadratic regression, R^2^=0.9998). All samples were analyzed in duplicate, and concentrations were automatically calculated from the fitted curve. Detailed cross-reactivity or analytical sensitivity information was not available in the hospital laboratory system.

### Statistical analyses

The Shapiro-Wilk test was used to test the normality of the data; Levene’s test was used for the homogeneity of variance assumption. Values were expressed as the mean (standard deviation [SD]), median (25–75th percentile), or n (%). Pearson’s chi-squared test or Fisher’s exact test was used to compare the groups. The student’s t-test or z-test and nonparametric comparisons were made using the Mann-Whitney U test. SPSS ver.18 (http://www.spss.com.hk/statistics/) was used for all analyses.

Post-hoc power was calculated with a two-sided independent-samples t-test (α=0.05) using the observed effect sizes and group sizes (PCOS n=82, control n=92). Effect size (Cohen’s d) was computed from the pooled within-group SD. The achieved power was 0.87 for P1NP (d=0.52), 0.93 for βCTX (d=0.58), and 0.88 for 25OH-D (d=0.52).

## RESULTS

A comparison of demographic characteristics between the groups is presented in Table 1. There was no significant difference between the PCOS and control groups in terms of age, ethnicity, nulliparity, smoking habits, weight, height, and BMI. As shown in Table 2, FSH (p=0.111), estradiol (p=0.569), TSH (p=0.922), prolactin (p=0.769), DHEA-S (p=0.306), total testosterone (p=0.237), fasting glucose (p=0.328), fasting insulin (p=0.086), HOMA-IR (p=0.091), or HbA1c (p=0.416) were observed to be similar. A significant difference was observed between the levels of biochemical parameters, namely LH (p<0.001), LH/FSH (p<0.001), 25OH-D_3_ (p=0.010), P1NP (p=0.004), and βCTX (p=0.013).

**Table 1:** Demographic characteristics of study participants.

Characteristic	Control group (n: 92)	PCOS group (n: 82)	p-value
Age (years)	23.33±3.97	24.00±4.43	0.459
Weight (kg)	62.85±10.04	61.41±8.96	0.263
Height (cm)	1.64±0.07	1.63±0.05	0.484
BMI (kg/m^2^)	23.41±3.76	23.25±3.26	0.836
Ethnicity (Caucasian)	80 (87.0)	72 (87.8)	0.801
Nulliparity	84 (91.3)	76 (92.7)	0.735
Smokers	26 (28.2)	20 (24.4)	0.281

PCOS: polycystic ovary syndrome; BMI: body mass index.

**Table 2:** Comparison of clinical, hormonal, and biochemical parameters between the groups.

Parameter	Control group	PCOS group	p-value
FSH (IU/L)	6.41±1.43	5.96±1.64	0.111
LH (IU/L)	6.16±2.92	8.98±4.12	**<0.001**
LH/FSH	0.95±0.34	1.54±0.67	**<0.001**
Estradiol (pg/mL)	35.95±19.76	37.13±17.49	0.569
TSH (mIU/L)	2.22±1.08	2.28±1.15	0.922
PRL (μg/L)	26.36±15.69	23.46±10.79	0.769
DHEA-S (μg/dL)	250.57±86.04	264.55±82.39	0.306
Total testosterone (ng/L)	0.39±0.17	0.42±0.15	0.237
Fasting glucose (mg/dL)	91.50±7.10	92.41±7.58	0.328
Fasting insulin (mIU/L)	10.19±4.44	11.72±5.73	0.086
HOMA-IR	2.32±1.06	2.68±1.33	0.091
HBa1c (%)	5.24±0.30	5.29±0.21	0.416
Vitamin D (ng/mL)	117.08±73.92	80.12±68.00	**0.010**
βCTX (ng/L)	929.91±641.38	572.89±585.95	**0.004**
P1NP (ng/mL)	863.48±509.01	597.36±501.50	**0.013**

PCOS: polycystic ovary syndrome; FSH: follicle-stimulating hormone; LH: luteinizing hormone; TSH: thyroid-stimulating hormone; PRL: prolactin; DHEA-S: dehydroepiandrosterone sulfate; HbA1c: hemoglobin A1c; βCTX: beta-C terminal telopeptide; P1NP: procollagen-1 N-terminal peptide; HOMA-IR: Homeostatic Model Assessment of Insulin Resistance. Bold values indicate statistically significant results (p<0.05).

Notably, 25OH-D_3,_ involved in bone metabolism, was 117.08±73.92 in the control group and 80.12±68.00 ng/mL in the PCOS group (p=0.010). The mean of bone turnover marker P1NP was 863.47±509.01 ng/mL in the control group and 597.36±501.49 ng/mL in the PCOS group (p=0.013). The mean βCTX was 929.91±641.38 ng/L in the control group and 572.88±585.94 ng/L in the PCOS group (p=0.014). As a result of the correlation with bone turnover markers, a strong correlation was observed between 25OH-D and βCTX (p≤0.001, r=0.944) and P1NP (p≤0.001, r=0.935). It was observed that there was a significant correlation among the bone turnover markers (Table 3).

**Table 3:** Correlation between bone turnover markers.

Parameter	Vitamin D		βCTX		P1NP	
	r	p-value	r	p-value	R	p-value
Vitamin D			0.944	**<0.001**	0.935	**<0.001**
βCTX	0.944	**<0.001**			0.936	**<0.001**
P1NP	0.935	**<0.001**	0.936	**<0.001**		

βCTX: beta-C terminal telopeptide; P1NP: procollagen-1 N-terminal peptide. Note: The very high correlations (r>0.93) likely reflect the between-group separation rather than individual biological coupling; therefore, they should be interpreted cautiously. Bold values indicate statistically significant correlations (p<0.05).

Vitamin D levels were measured in ng/mL using the BT-LAB E1543Hu ELISA kit (range 0.5–200 ng/mL according to the manufacturer). The relatively high mean value observed in the control group likely reflects kit-related variability and the use of vitamin D supplements by several participants. Since both groups were analyzed using the same assay under identical laboratory conditions, the absolute values are less clinically relevant than the relative difference between groups.

## DISCUSSION

PCOS is the most common endocrine–metabolic disorder in women of reproductive age. Hormonal dysregulation, chronic ovulatory dysfunction, and underlying low-grade inflammation may adversely influence bone turnover and BMD. In this study, we assessed three key parameters of bone metabolism: the bone formation marker P1NP, the bone resorption marker βCTX, and 25OH-D, which plays a central role in calcium and bone homeostasis. Our findings showed that all three markers were significantly lower in non-obese women with PCOS compared with controls, suggesting that PCOS may be associated with a state of reduced bone turnover independent of obesity.

### Results from previous studies

The effects of estrogens and androgens on bone metabolism are obvious. In PCOS, alterations in bone turnover and BMD are influenced by the underlying hormonal milieu. Recent evidence highlights that multiple interconnected mechanisms—including chronic low-grade inflammation, oxidative stress, vitamin D deficiency, reduced growth hormone secretion, hyperinsulinemia, and hyperandrogenemia—can impair bone formation and remodeling^8,9^. Large-scale studies and systematic reviews published after 2020 confirm that chronic subclinical inflammation is a consistent feature of PCOS pathophysiology, driving oxidative stress and contributing to compromised bone quality and altered bone turnover^10,11,12^. These inflammatory and oxidative pathways appear to mediate detrimental effects on both trabecular and cortical bone compartments.

Several studies have sought to clarify the effects of PCOS on bone health, yet results remain inconsistent. A recent narrative review reported that, in some cohorts, BMD differences between women with PCOS and BMI-matched controls are minimal, suggesting that adiposity may mitigate potential skeletal consequences of the syndrome and that chronic low-grade inflammation alone may not significantly alter bone physiology^9^. In their study investigating plasma growth and differentiation factor (GDF)-9 and GDF-15 levels in PCOS and their possible relationships with bone turnover parameters and BMD, Berberoglu et al. found a significant correlation between GDF-15 concentrations and both age and the homeostasis model assessment index, but no significant differences were observed in bone parameters between the PCOS and control groups. They have also shown that there was no significant difference in the femoral neck, trochanter, total hip, and lumbar BMD between the groups^6^. Kassanos et al. have evaluated cortical BMDs in the control group and obese and normal PCOS patients^13^. This was the first study to use peripheral quantitative computed tomography to evaluate previously reported parameters of the distal tibia in PCOS patients and a healthy control group. They have found that trabecular bone density, geometric parameters of bone, bending resistance, and torsional strength increase in PCOS women, but the difference was not significant. On the other hand, cortical bone density significantly increases not only in obese PCOS women but also in lean PCOS patients and has shown increases in BMD in PCOS patients^13^.

In a previous study evaluating bone formation markers (osteocalcin and bone-specific alkaline phosphatase) and bone resorption markers (urinary deoxypyridinoline and pyridinoline) in obese women aged 25–35 years, no significant differences were observed between PCOS and control groups^14^. More recent evidence supports the age- and BMI-specific nature of skeletal changes in PCOS. A 2022 cross-sectional study found that serum P1NP, βCTX, and 25OH-D levels were significantly lower in non-obese women with PCOS aged 20–35 years compared with BMI-matched controls, suggesting that the absence of excess adiposity may unmask adverse effects of PCOS on bone turnover^9^. These findings are consistent with the present study, which similarly included only non-obese women aged 18–35 years and found comparable reductions in these bone-related parameters.

Evidence regarding fracture risk in women with PCOS remains conflicting. Some large population-based studies, such as that by Rubin et al. in 76,682 Danish women, have reported a reduced fracture risk among women under 30 years of age with PCOS^15^, whereas a nationwide Taiwanese cohort study found an increased fracture risk in 11,606 women with the syndrome^16^. More recently, a 2024 meta-analysis by Piovezan et al. showed that non-obese women with PCOS (BMI<27 kg/m^2^) exhibit significantly lower spinal and femoral bone mass compared with BMI-matched controls, while this reduction is not observed in obese women^17^. These findings suggest that differences in adiposity, hormonal milieu, and bone turnover rates may partly explain the heterogeneity in fracture outcomes. Reduced bone turnover can help preserve bone density in older age groups, whereas high bone turnover may accelerate bone loss and compromise bone quality^18,19^.

Previous studies have demonstrated that lean women with PCOS exhibit lower spinal and femoral BMD, whereas obese PCOS patients often show preserved or increased bone mass compared with BMI-matched controls^8,17^. These results are consistent with our biochemical findings and support the concept that obesity may mitigate bone loss in PCOS.

### Clinical implications

Our findings have potential implications for routine clinical practice. Serum 25OH-D3, a key determinant of bone metabolism in PCOS, was reduced in our non-obese cohort, consistent with previous studies linking lower 25OH-D3 levels to increased BMI and confirming this association in PCOS populations^20^. In addition, recent systematic reviews have emphasized the broader metabolic and reproductive impact of vitamin D in PCOS, further supporting its relevance as a biological modulator in this population^21^. Moreover, 25OH-D3 correlated strongly with bone turnover markers. Both P1NP (bone formation) and βCTX (bone resorption) were significantly decreased in the PCOS group, aligning with the results of Lingaiah et al. in women with PCOS under 30 years of age^22^. The concurrent reduction in bone formation and resorption may indicate a compensatory mechanism within bone metabolism. Clinically, these findings highlight the need to inform patients at diagnosis regarding potential changes in bone health. These findings suggest that non-obese women with PCOS may benefit from early vitamin D supplementation, nutritional optimization, and regular lifestyle interventions. In patients with additional risk factors, bone density screening may also be advisable. Notably, 25OH-D supplementation should be considered where appropriate, and women with a family history of fragility fractures or a long-standing PCOS diagnosis should be prioritized for early screening using DEXA and bone turnover markers.

The simultaneous decrease in both bone formation (P1NP) and bone resorption (βCTX) may reflect a state of low bone turnover. This could be due to hormonal dysregulation, chronic inflammation, oxidative stress, and vitamin D deficiency, which impair both osteoblast and osteoclast activity^17^. Although a low-turnover state may preserve bone mass in the short term, chronically reduced remodeling can impair bone quality and limit the repair of microdamage. Therefore, the long-term clinical implications of low bone turnover in nonobese women with PCOS remain unclear and warrant further prospective investigation.

### Strengths and limitations

The present study possesses notable methodological strengths. First, all blood samples were obtained on the third day of the menstrual cycle, thereby minimizing intra-cycle hormonal fluctuations. Second, participants underwent an overnight fast of at least 8 h prior to sampling, eliminating postprandial variability in βCTX measurements. Third, individuals with known hepatic or renal disorders were excluded, given the role of these organs in βCTX and P1NP clearance. Finally, restricting the cohort to non-obese women aged 18–35 years minimized confounding effects related to adiposity and advanced age. However, several limitations should be acknowledged. Seasonal variation in 25(OH)D levels could not be assessed because the timing of blood sampling was not recorded. Data on physical activity, a known determinant of bone metabolism, were also not obtained. Moreover, participants were not stratified according to PCOS phenotype (hyperandrogenic vs. non-hyperandrogenic), which may influence hormonal and metabolic characteristics^23^. Future studies incorporating phenotype-based subgroup analyses are warranted to clarify potential differences in bone health across PCOS subtypes.

## CONCLUSION

Our results indicated that PCOS adversely affects bone turnover in non-obese patients. In addition, an additional prospective study using women from reproductive age to menopause, in which DEXA and BMD measurements are taken together, will provide more accurate results. Our findings suggest that bone metabolism may be compromised even in non-obese women with PCOS. Early assessment of bone health and vitamin D supplementation should be considered in PCOS management. Future studies should stratify participants by PCOS phenotype to clarify potential differences in bone metabolism across subtypes.

## Data Availability

The datasets generated and/or analyzed during the current study are available from the corresponding author upon reasonable request.
